# Trace Elements Open a New Direction for the Diagnosis of Atherosclerosis

**DOI:** 10.31083/j.rcm2401023

**Published:** 2023-01-11

**Authors:** Heyu Meng, Jianjun Ruan, Yanqiu Chen, Zhaohan Yan, Jinsha Liu, Xue Wang, Xin Meng, Jingru Wang, Qiang Zhang, Xiangdong Li, Fanbo Meng

**Affiliations:** ^1^Department of Cardiology, China-Japan Union Hospital of Jilin University, 130033 Changchun, Jilin, China; ^2^Jilin Provincial Precision Medicine Key Laboratory for Cardiovascular Genetic Diagnosis, Jilin Provincial Cardiovascular Research Institute, Jilin University, 130033 Changchun, Jilin, China; ^3^Jilin Provincial Engineering Laboratory for Endothelial Function and Genetic Diagnosis of Cardiovascular Disease, Jilin Provincial Cardiovascular Research Institute, Jilin University, 130033 Changchun, Jilin, China; ^4^Jilin Provincial Molecular Biology Research Center for Precision Medicine of Major Cardiovascular Disease, Jilin Provincial Cardiovascular Research Institute, Jilin University, 130033 Changchun, Jilin, China

**Keywords:** trace elements, metal trace elements, nonmetallic trace elements, atherosclerosis, obesity, diagnosis, risk factors

## Abstract

Abnormal or excessive accumulation of adipose tissue leads to a condition called obesity. Long-term positive energy balance arises when energy intake surpasses energy expenditure, which increases the risk of metabolic and other chronic diseases, such as atherosclerosis. In industrialized countries, the prevalence of coronary heart disease is positively correlated with the human development index. Atherosclerotic cardiovascular disease (ACD) is among the primary causes of death on a global scale. There is evidence to support the notion that individuals from varied socioeconomic origins may experience varying mortality effects as a result of high blood pressure, high blood sugar, raised cholesterol levels, and high body mass index (BMI). However, it is believed that changes in the concentration of trace elements in the human body are the main contributors to the development of some diseases and the transition from a healthy to a diseased state. Metal trace elements, non-metal trace elements, and the sampling site will be examined to determine whether trace elements can aid in the diagnosis of atherosclerosis. This article will discuss whether trace elements, discussed under three sections of metal trace elements, non-metal trace elements, and the sampling site, can participate in the diagnosis of atherosclerosis.

## 1. Introduction

The rise in blood cholesterol and blood sugar levels due to dietary imbalance, as well as unhealthy behaviors like smoking and genetic factors, are all contributing causes to the rise in patients with coronary heart disease in the United States, Europe, and China [1]. The four main risk factors for coronary heart disease are smoking, blood sugar, blood lipids, and hypertension. The aforementioned four independent risk factors were found to be the primary indicators for coronary atherosclerosis [2, 3, 4]. Moreover, it has been found that the frequency of coronary heart disease in underdeveloped countries was positively correlated with the human development index. Contrarily, it has a negative correlation (*p* = 0.47 and 0.34, respectively) with the human development index of wealthy nations. Due to dietary imbalances and variations in the concentration of trace elements in serum, coronary heart disease incidence rates have changed over the past few decades, with increases in developing nations and decreases in industrialized countries (*p* = 0.021 and 0.002, respectively) [5, 6, 7, 8, 9]. According to a recent Lancet study, there is a link between obesity and several diseases [10, 11, 12]. A thorough understanding of the effects of obesity on health is provided by the simultaneous evaluation of 78 disease outcomes [12, 13, 14]. Numerous organ systems are affected by 21 non-overlapping disorders that are related to severe obesity (HR ≥1.50, *p *< 0.0006) [12]. These disorders are interrelated to the level that individual obesity-related disorders can be predicted using one or more obesity-related diseases [15]. Secondly, this association accelerates the rate at which multiple obesity-related incidents occur. In the Finnish cohort, obese people had a 5-fold higher risk of simple multiple morbidities and a 12-fold higher risk of complicated multiple morbidities than those who were of a healthy weight [16]. Among obese participants under the age of 50, the risk of complex multiple incidence rate is higher than that of elderly obese patients. According to the classification of obesity, the relative risk of complex and common diseases increases with increasing levels of obesity [17, 18]. Third, there are 140 possible combinations of 21 diseases in the overall pattern of complex multiple incidence rates associated with obesity, which is highly varied (cardiometabolic, digestion, respiration, nerves, musculoskeletal, infection, and malignant diseases). Despite having a high incidence rate, obesity is only mildly linked to death, suggesting that it affects overall survival less than disease-free survival. These three conclusions were supported by a separate cohort of senior people from the UK Biobank [14, 15, 16, 17, 18].

These days, lifestyle, stress, nutrition, genetics, and a lack of exercise are all contributing to an increase in obesity among the world’s population. White adipose tissue (WAT) in obese people not only stores extra energy but also disrupts endocrine function. WAT secretes a class of chemicals known as adipokines, which have endocrine, autocrine, and paracrine functions in the central nervous system (CNS) and throughout the body [19, 20, 21]. Obesity increases the chance of developing atherosclerosis and Alzheimer’s disease (AD) [22, 23]. Obesity has been identified as a risk factor for coronary heart disease. Additionally, obesity can cause low-grade chronic inflammation of adipose tissue, which upsets the homeostasis system and brings on a variety of disorders, including those associated with neurodegeneration. Interleukin-1β (IL-1β), Interleukin-6 (IL-6), tumor necrosis factor α (TNF-α), and leptin are examples of proinflammatory adipokines produced by adipose tissue during this process, while anti-inflammatory adipokines such adiponectin are decreased [24]. These processes are thought to play a part in the development of atherosclerosis, which has also been supported by our earlier research [24, 25, 26, 27].

Obesity and diabetes are both complex, multifaceted disorders that can often be prevented [28, 29]. Diabetes also considerably raises the risk of coronary atherosclerosis [30]. The leading cause of death worldwide is atherosclerotic cardiovascular disease (ACD) [31]. Risk factors including high blood pressure, diabetes, high cholesterol, and body mass index (BMI) have different long-term effects on patient groups with different income levels in terms of mortality [5, 32, 33]. Over the past 20 years, high-income countries have been able to minimize the consequences of these risk factors, while low- and middle-income countries have experienced an increase in mortality as a result of high BMI and blood sugar levels [33]. The rise in atherosclerosis mortality is attributed to several factors, including population growth and aging, considerable nutritional system changes, and population growth [34]. However, it is uncertain whether differences in the dietary intake of trace elements contribute to coronary atherosclerosis [35, 36].

According to the reported studies, coronary heart disease and several other disorders are closely associated to trace elements in the human body [37, 38]. According to studies by Zang *et al*. [39], metal levels in the blood and obesity in children and adolescents are positively correlated. It has been discovered that obesity is associated with an increase in superoxide dismutase (SOD) and total circulation copper concentration. Metal ions affect leptin production in adipocytes by regulating the release of free fatty acids and the consumption of glucose, highlighting that obesity is a substantial risk factor for coronary heart disease [39, 40, 41]. Gonzalez *et al*. [42]. revealed that acute coronary syndrome (ACS) is the outcome, not the cause, of the iron saturation of the iron transporter transferrin (transferrin saturation: TSAT). TSAT is a crucial factor in the diagnosis of ACS and is not up-regulated in the acute inflammatory response [42, 43]. Changes in trace elements can help type 2 diabetic patients with their insulin resistance, according to Kalita *et al*. [44]. It is known that the cofactors magnesium and manganese are necessary for multiple enzymes involved in diabetes. Low magnesium and manganese levels increase the risk of developing metabolic syndrome, which disrupts glucose metabolism. Atherosclerosis may be brought on by low levels of total glycerides (TG) and total cholesterol as well as magnesium deficiencies [44, 45]. According to Li *et al*. [46], blood selenium concentration was strongly related to both men’s and women’s all-cause mortality, although it was more significant in females with coronary heart disease. Hence, it is believed that the major cause of the development of various diseases and the transformation from a healthy condition to a diseased state is a change in the concentration of trace elements in the human body. Numerous studies have demonstrated the links between coronary heart disease and factors including smoking, high blood pressure, high blood sugar, and high cholesterol.

**Fig. 1.**
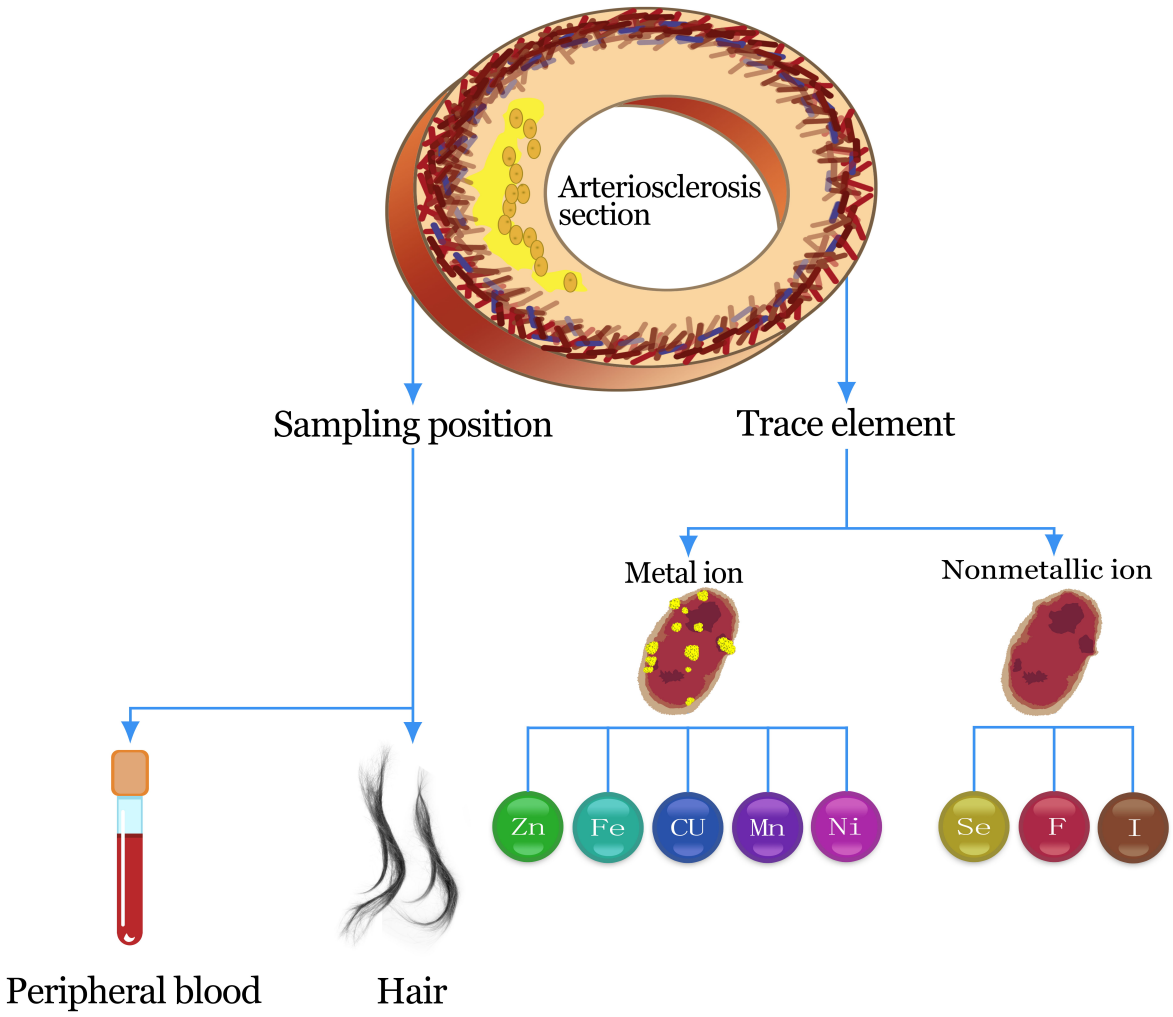
**Flow chart**. The process of atherosclerosis, the sampling location, and the significance of different types of trace elements for atherosclerosis.

There is a definite relationship between trace elements and atherosclerosis. This review will assess, from the perspective of sample location and the presence of specific trace elements, whether trace elements can serve as a novel indicator for detecting atherosclerosis (see Fig. 1 for details).

## 2. Sampling Position

**Fig. 2.**
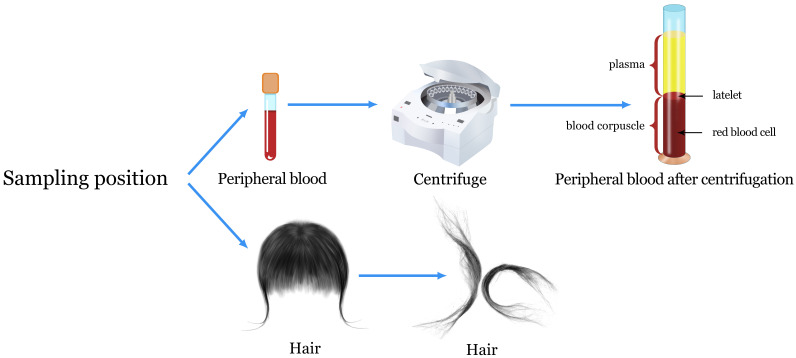
**Sampling details of samples**. Obtaining stratified centrifuged blood from the patient’s peripheral blood and using centrifuged serum is one method. Another method is to collect samples from the patient’s scalp and hair.

In this review, the advantages and disadvantages of peripheral blood and epidermal tissue have been compared [47]. See the subordinate part and Fig. 2 for details.

### 2.1 Peripheral Blood

Peripheral blood can be used as a window to evaluate diseases [48, 49, 50]. It is convenient for sampling and causes minimal harm [51]. The peripheral blood is separated into three layers following centrifugation: the upper plasma layer, the middle white blood cell layer, and the bottom red blood cell layer. Furthermore, the peripheral blood is separated into plasma and serum based on whether or not it has been anticoagulated. The upper layer obtained following anticoagulant treatment and centrifugation is plasma. Without anticoagulant treatment, the upper layer obtained following centrifugation is serum.

#### 2.1.1 Application of Leukocytes

Leukocytes in peripheral blood are one of the most crucial elements for diagnosing and assessing disease [52]. Samples of patients’ peripheral blood smears are analyzed by pathologists for clinical diagnosis. This examination is predicated mostly on the morphological properties of leukocytes and their nuclei and cytoplasm, such as their form, size, color, texture, maturity, and staining [53]. Leukocytes are closely associated with blood diseases and various other disorders. Recently, computer-aided diagnosis technology has developed rapidly in the field of digital hematology, which is related to the detection of leukocytes and their nuclei and cytoplasm, as well as segmentation and classification technology. These technologies continue to play a crucial role in digital hematological image analysis by delivering traceable clinical data and decreasing human intervention [54].

#### 2.1.2 Application of Serum

It has been suggested that serum proteins contribute to every aspect of life. Moreover, it has been reported that the human serum proteome is under strong genetic control [55]. Additionally, it was discovered that serum proteins are part of the regulatory group of network modules, which includes members synthesized in all parts of the body. The underlined finding suggests that thousands of proteins in the blood play a significant role in mediating coordination or homeostasis at the system level [56]. Importantly, deep serum and plasma proteomes are associated with a variety of diseases, human life spans, and the ability to predict how well individuals would respond to treatment [55, 57, 58, 59, 60, 61, 62, 63]. Recent research has linked genetics, protein levels, and diseases as well as highlighted the potential causative link between proteins and complex disorders [64, 65].

### 2.2 Epidermal Tissue

Elemental analysis of hair is a valuable diagnostic tool for assessing mineral nutrition in the body [66, 67]. The hair follicle is one of the most active metabolic tissues. Most metallokeratins have a high affinity for the sulfhydryl groups of amino acids, while melanin in hair conveniently binds cations through ionic interaction. The keratin outer layer of hair forms a barrier with the environment to prevent the escape of accumulated substances (ions). Hair analysis has become a popular source of information in the therapeutic, nutritional, toxicological, and forensic fields recently due to advancements in research techniques [68, 69]. Standard analytical procedures for determining the concentration of trace elements in blood and urine may not accurately reflect the current availability of biological elements in organisms. For example, the concentration of biological components in hair samples is significantly higher than that in serum, with zinc ion levels over one hundred times higher. As a result, serum metal concentrations do not accurately reflect total trace element concentrations in the body [70, 71]. Metal is implanted into the structure of hair continuously as it develops. Given that the average rate of growth in humans is 1 cm per month [72], the concentration of a given element in the hair represents the organism’s average long-term concentration. The metal concentration in the routine analysis sample decreases in a few days (blood) or weeks (urine); therefore, hair analysis is more valuable for assessing the long-term concentration of specific metals. For example, the sample examined is 3–4 cm long, so the analysis results show the average concentration of mineral nutrients in the past 3–4 months [72]. Such a large time window is rare for samples that can be easily obtained from patients. The results of elemental hair analysis are not affected by short-term changes in metal serum concentrations.

## 3. Trace Elements

Trace elements are substances comprising between 0.01% and 0.005% of our body weight. Trace elements have several physiological and biological applications. They may serve as hormones and vitamins, as well as primary or secondary components of biological macromolecules. Furthermore, they play a crucial role in keeping the body functioning normally. Among these, critical trace elements such as iron, copper, zinc, cobalt, chromium, manganese, and selenium are vital components in the body. The body is unable to generate or synthesize the aforementioned substances, hence food must be consumed to meet these needs. Deficiency can easily occur if the diet is not properly balanced, is partial, or the sufferer is ill. These elements all play a role in the development of atherosclerosis [73]. Trace elements play a significant influence on the status of cardiovascular disease because they directly or indirectly affect circulatory processes [74, 75, 76].

### 3.1 Metal Ions

#### 3.1.1 Zinc

Zinc ions influence the body’s metabolism of a variety of proteins, lipids, and carbohydrates as well as a variety of other cellular metabolic activities [77, 78]. More than 70 enzymes depend on zinc, including glutathione peroxidase and superoxide dismutase. By serving as a cofactor of Cu-Zn superoxide dismutase (Cu, Zn-SOD), zinc can have an impact on CHD (coronary heart disease). According to the reported studies, zinc supplementation decreases Cu-Zn superoxide dismutase activity because copper absorption and increased zinc consumption have an antagonistic relationship [79]. Moreover, zinc also has anti-inflammatory and antioxidant effects [80, 81]. With the increase in zinc concentration, cells are better able to act as antioxidants, and normal endothelium function is maintained. The role of zinc in enzymes, humoral mediators, and mitosis is crucial for the function of the immune system [82]. Zinc deficiency can lead to an increase in oxidative stress sensitivity, as well as interleukin-1 and tumor necrosis factor α increase of factors. The expression is connected to endothelial cells’ enhanced apoptosis.

The zinc ion concentration of female patients with coronary heart disease was low, according to our study of 3541 cases [83]. Further grade analysis revealed a connection between the decline in menopausal hormones in women and the decline in zinc ion concentration. It was discovered that the zinc ion concentration of female smokers over 50 years of age was different, using the age of 50 as the cutoff threshold for hormone secretion drop [83]. It, therefore, makes sense to speculate the association between the decrease in hormone secretion during female menopause, the concentration of zinc, and the occurrence of coronary heart disease in menopausal women.

#### 3.1.2 Iron

Iron holds critical importance for a variety of physiological processes. Proteins and enzymes that contain iron are crucial components of cell metabolism. These enzymes and proteins are necessary for mitochondrial function, DNA synthesis, DNA repair, cell death, and cell proliferation [84, 85, 86]. Additionally, the generation of red blood cells (RBCs) and the transportation of oxygen both depend on iron, which is the primary component of hemoglobin. Due to its propensity to form reactive oxygen species (ROS) and its ability to catalyze the Fenton reaction, which produces hydroxyl radicals, iron may also be toxic at high doses [87]. In addition, it is essential for determining the amount of bacterial toxicity [88].

The disease may also result from irregular iron intake or output. Initially, iron was linked to the onset of coronary atherosclerosis [87, 89]. LDL oxidation can be hastened by free iron [90]. Due to the absorption of low-density lipoprotein by macrophage LDL receptors, foam cells congregate. The two primary steps in the development of coronary atherosclerosis are the development of a necrotic core and the invasion of foam cells [91]. Numerous macrophage subtypes have been identified in atherosclerotic plaques [92]. Macrophages are crucial to the development of coronary atherosclerosis. The primary cause of M1 macrophage activation in plaques is lipid absorption, which can result in foam cell formation and the generation of inflammatory cytokines [93]. It is postulated that M1 macrophages produce coronary atherosclerosis through media-induced paracrine stimulation of MSC (mesenchymal stem cells) migration and proliferation in the intima.

Plaque instability may result from the M1 cells’ production of a fibrous cap after the hydrolysis of collagen fibers [94]. Additionally, M2 macrophages are stimulated by Th2 cytokines including IL-4, IL-10, and IL-13 to create anti-inflammatory cytokines. The inflammatory response is balanced by M2 macrophages, which also aid in tissue healing and inflammation reduction. The M1/M2 model provides a concise framework for comprehending macrophage behavior in a damaged environment. Although M2 macrophages can export and metabolize iron, M1 macrophages have an advantage in iron accumulation due to their high ferritin level. Changes in the iron turnover between M1 and M2 macrophages may contribute to coronary atherosclerosis. The association between peripheral blood iron concentration and coronary atherosclerosis was validated by a cross-sectional study encompassing more than 4000 individuals [95]. A biomarker for the prediction of coronary atherosclerosis is the decrease of iron ions in peripheral blood [95].

#### 3.1.3 Copper

Copper is the most abundant element in the human brain [96, 97, 98] and is involved in a variety of biological functions. Its abnormal changes have been linked to numerous neurological diseases [99, 100]. In biological systems, copper ions usually have coexisting oxidation states. On the other hand, copper has various complexes, including distribution, storage, and transportation. Therefore, in a diseased condition [101], the dynamic change in copper concentration results in abnormal accumulation or uncontrollable oxidation-reduction reactions [102], which then disturb the natural balance or start a chain of events that eventually result in neurodegenerative diseases like Alzheimer’s and Parkinson’s [103]. To understand how copper changes over time, we need to make more copper detection tools, especially ones that can accurately separate copper ions without damaging samples.

With the development of a large number of ${\mathrm{Cu}}^{2+}$ detection methods [104, 105], fluorescent probe methods have received widespread attention due to their probe characteristics, such as visible fluorescent signals and immediate response to targets [106, 107, 108]. In particular, the rhodamine spiral CT (computed tomography) ${\mathrm{Cu}}^{2+}$ probe, its significant copper ion evaluation, and the specific color change accompanying the recognition process are particularly prominent. Efforts are being made to improve the probe’s sensitivity and selectivity to ${\mathrm{Cu}}^{2+}$ in complex environments or biological systems [109, 110, 111], the probe’s long-wavelength excitation and emission characteristics [112], thereby avoiding biological interference and the probe’s solubility and affinity [113].

#### 3.1.4 Manganese

Manganese (Mn), an essential component of the human body, is mostly obtained through food and water. Mn enters the body via the digestive system and is then transported to tissues with a high concentration of mitochondria, such as the pituitary, liver, and pancreas, where it is rapidly accumulated [114, 115]. The synthesis and activation of numerous enzymes, including oxidoreductases, transferases, hydrolases, lyases, isomerases, and ligases, as well as glucose and lipid metabolism, the accelerated synthesis of protein, vitamin C, and vitamin B, the catalysis of hematopoiesis, the regulation of the endocrine system, and the enhancement of immune function, are all facilitated by Mn [116, 117]. Other Mn metalloenzymes, such as arginase, glutamine synthetase, phosphoenolpyruvate decarboxylase, and manganese superoxide dismutase (MnSOD), also help with these metabolic processes and lessen the oxidative stress on free radicals.

In recent years, there has been a marked rise in the incidence of metabolic illnesses like type 2 diabetes (T2DM), obesity, insulin resistance, atherosclerosis, hyperlipidemia, nonalcoholic fatty liver disease (NAFLD), and hepatic steatosis [118]. Metabolic syndrome is typically what causes these metabolic illnesses (MetS). MetS criteria must be met if three of the five markers are present: abdominal obesity, impaired glucose metabolism, hypertension, and dyslipidemia, which includes higher triglyceride levels and reduced high-density lipoprotein (HDL) levels [119]. Additionally, several studies [120, 121, 122, 123, 124, 125, 126] have demonstrated a connection between oxidative stress and inflammation, and metabolic disorders. Mn is a component or activator of various enzymes, primarily antioxidants, and it is crucial for maintaining appropriate insulin production and secretion as well as glucose and lipid metabolism. As a result, manganese may prevent the development of MetS [127].

It’s important to note that Mn is required for MnSOD (Manganese Superoxide Dismutase) to lower mitochondrial oxidative stress. ROS are produced mostly by mitochondria in both healthy and diseased cells. Various neuropathological illnesses are associated with the increased production of glucocorticoids, which are crucial in controlling the biosynthesis and metabolism of proteins, lipids, and carbohydrates. Excessive ROS inappropriately accumulates and causes oxidative damage [128]. MnSOD is a significant antioxidant that can also remove superoxide produced in mitochondria and guard against oxidative stress [129, 130]. If mitochondria are damaged or dysfunctional, the production of ROS and oxidative stress are exacerbated [131].

According to a reported study, central obesity, increased triglycerides, decreased high-density lipoprotein cholesterol, elevated blood pressure, and elevated fasting glucose is the five factors that often define MetS [132]. The existence of MetS aids in the identification of high-risk people who have T2DM and cardiovascular disease (CVD) [132]. A frequent risk factor for MetS components is oxidative stress. In MetS patients, persistent low-level inflammation and oxidative stress state are typically regarded as second-level abnormalities, while insulin resistance is typically regarded as the first level of metabolic alterations [133]. All individual MetS components and the incidence of cardiovascular problems in MetS participants are linked to oxidative stress [133, 134, 135, 136].

#### 3.1.5 Nickel 

One of the key elements used to gauge air quality is nickel. About 20% of people have an allergy to nickel ions, and there are more female patients than male patients. Nickel is the most frequently sensitizing metal. Nickel ions can enter the skin through pores and sebaceous glands and cause skin allergy and inflammation, which are clinically seen as dermatitis and eczema when in touch with the human body [137]. Nickel allergy frequently lasts forever once sensitization takes place. Humidity, pressure, sweat, and friction can exacerbate nickel allergy symptoms. Nickel-allergic dermatitis manifests clinically as itchy, papular, or vesicular dermatitis with mossing [138]. In nickel deficiency, the activity of six dehydrogenases decreases, including glutamate dehydrogenase, glucose-6-phosphate dehydrogenase, lactate dehydrogenase, and isocitrate dehydrogenase. These enzymes are essential for the tricarboxylic acid cycle, anaerobic glycolysis, the synthesis of NADH (nicotinamide adenine dinucleotide), and the release of nitrogen from amino acids. Nickel deficiency was also found to change the structure of hepatocytes and mitochondria, especially with an irregular endoplasmic reticulum and a drop in oxidative activity [139].

In a study on the Chinese population, the link between several chemical elements, pollution sources for ambient fine particles (PM2.5), and oxidative stress indicators in healthy college students was examined. Participants in the study underwent 12 additional blood draws before and after transferring from suburban to urban campuses in Beijing, China, which had significant levels of air pollution. The 95% confidence interval (CI) of ox LDL is thought to widen with each quartile point rise in nickel (2.5 ng/$m^{3}$) and with the duration of exposure to polluted air [140, 141, 142].

### 3.2 Non-Metallic Ions

#### 3.2.1 Selenium

Selenium (SE) is a crucial part of selenoprotein and an essential element for antioxidant defense. The involvement of selenium in antioxidant defense, which is mediated by the glutathione peroxidase (*GPX*) family, supports the nutrient’s preventive action against dyslipidemia and cardiovascular disease (CVD). In this case, *GPX* slows or stops atherosclerosis by lowering the production of hydroperoxides of phospholipids and cholesterol esters and stopping the buildup of oxidized low-density lipoprotein (LDL) in arteries [143, 144, 145].

Low activity of this enzyme will harm the body’s antioxidant defense system because of insufficient selenium intake and the presence of *GPX1* gene polymorphism [146]. According to several studies, single nucleotide polymorphisms (SNPs) of the *GPX* gene have been linked to a higher risk of metabolic syndrome and cardiovascular disease [147, 148]. Due to the lack of extensive studies, the cardioprotective effects of selenium are currently debatable [149].

Recent research has demonstrated that patients with CVID (common variable immunodeficiency) had considerably lower levels of plasma selenium and *GPX* activity than healthy controls [150]. Se level, apolipoprotein A-1 concentration, and the main proteins that make up the high-density lipoprotein cholesterol component all showed a substantial positive association. Numerous bacteria are capable of producing selenocysteine, suggesting that selenoproteins may be important for bacterial physiology [151]. Moreover, dietary selenium impacts the composition of the host microbiota. Therefore, pathogenic bacteria, microbiota, and host immune cells may dispute the limited availability of selenium, which is especially critical in CVID patients [151, 152, 153]. Se and apoA-1 concentrations are significantly correlated in the literature. In predicting the risk of cardiovascular disease, apolipoprotein A-1 and other HDL-C functional markers outperform HDL-C concentration [154].

#### 3.2.2 Fluorine

In previously reported studies [155], anatomical imaging models used to measure atherosclerosis usually focused on non-localization characteristics. The degree of luminal stenosis can be examined using conventional ultrasound and angiography methods, and coronary calcium scores can be generated using cardiac CT to measure coronary artery calcification. However, alternative techniques have started to be used with the explicit aim of identifying plaque components, such as multi-detector CT coronary angiography, MRI, intravascular ultrasound (IVUS), and optical coherence tomography [155, 156]. Since these patterns are based on anatomy and don’t have the sensitivity and chemical specificity of early detection, they are best suited for revealing late structural changes or calcifications in the vessel wall [157].

In contrast to anatomical imaging, molecular imaging mode can identify minute processes like microcalcification and inflammation. These chemical alterations take place before the aforementioned morphological changes during the early stages of the disease process. The most well-known of these molecular technologies is positron emission tomography (PET), which uses the radiotracer 18F fluorodeoxyglucose (18F-FDG), a radiolabeled glucose analog that is used as a marker of metabolic activity and, in turn, for inflammation. In contrast, 18F sodium fluoride (18F NaF), a particular marker of bone mineralization, has lately been utilized to diagnose vascular calcification after being traditionally used to diagnose metastatic bone malignancy.

One of the initial molecular markers used to assess atherosclerosis was 18F-FDG, which is currently the most widely used PET radiotracer and may also be the most thoroughly researched. Early research supported the use of 18F-FDG as a diagnostic tool by demonstrating a relationship between the vascular system’s 18F-FDG activity and atherosclerosis and cardiovascular risk factors [158, 159, 160, 161]. More concrete proof of the connection between 18F-FDG uptake and atherosclerotic disease was found in later investigations. 18F-FDG activity is higher in 58–85% of carotid lesions, according to studies on patients with a history of cerebrovascular accidents [162, 163]. Before endarterectomy, Tawakol *et al*. [164] used PET scanning on patients with severe carotid stenosis and discovered a strong relationship between the uptake of 18F-FDG in carotid plaques and the staining of macrophages on comparable pathological specimens. Rudd *et al*. [165] utilized autoradiography to show in a related investigation that 18F-FDG avid lesions in individuals with symptomatic carotid stenosis were associated with macrophage-rich plaque regions in endarterectomy specimens. Additionally, the researchers discovered that symptomatic carotid lesion uptake of 18F-FDG was 27% higher than ipsilateral asymptomatic lesions [165].

The use of 18F-FDG PET imaging to evaluate plaque vulnerability and the risk of associated ischemia episodes has been supported by some evidence from other research [166]. Plaques with high-risk morphological characteristics on CT and histology exhibit a much higher affinity for 18F-FDG, according to Figueroa *et al*. [167]. Patients with elevated arterial 18F-FDG uptake have been observed to be more prone to experiencing ischemic cardiovascular events or cerebrovascular events [168, 169].

#### 3.2.3 Iodine

Iodine is commonly found in food, water, and iatrogenic sources, and excessive intake can result in disorders such as goiter [170, 171]. Iatrogenic iodine excess is the most prevalent adverse pharmaceutical effect, with amiodarone serving as a typical example [172]. Goiter, hyperthyroidism, hypothyroidism, and autoimmune thyroid disorders can all result from excessive iodine intake in humans [173, 174]. Additionally, some investigations have revealed that too much iodine can harm intellectual growth [175]. Furthermore, the effects of iodine excess on the cardiovascular system are still largely unknown.

According to an epidemiological analysis, individuals in the high-water iodine area had a greater incidence rate and more severe carotid atherosclerosis than adults in lower-water iodine locations [176]. Excessive iodine may harm rat aortic endothelial cells, according to cell tests [177]. The carotid artery is the area of atherosclerosis that is most susceptible and is a sensitive sign of vascular disorders. Carotid intima-media thickness (IMT) is a predictor of cardiovascular events [178] and a marker of atherosclerosis in coronary arteries and other blood vessels [179, 180, 181].

## 4. Conclusions

Taken together, trace elements are closely related to atherosclerosis and play different roles in different stages of atherosclerosis. In the prevention stage, reducing the intake of gases contaminated with nickel ions and foods containing manganese ions can reduce the occurrence of atherosclerosis. Meanwhile, foods containing selenium are protective factors for atherosclerosis. Animal studies have demonstrated that iodine ions can impair aortic endothelial cells, hence it is recommended to limit their consumption. If atherosclerosis is already present, early atherosclerotic calcification can be found by using probes with copper ions and 18F sodium fluoride (18F NaF) in the early stages of the disease. Detecting the contents of serum zinc and iron ions in the treatment stage is helpful to evaluate the recovery from atherosclerosis.
